# Multi-Strategy Bald Eagle Search Algorithm Embedded Orthogonal Learning for Wireless Sensor Network (WSN) Coverage Optimization

**DOI:** 10.3390/s24216794

**Published:** 2024-10-23

**Authors:** Haixu Niu, Yonghai Li, Chunyu Zhang, Tianfei Chen, Lijun Sun, Muhammad Irsyad Abdullah

**Affiliations:** 1 Faculty of Information Science and Engineering, Management and Science University, Shah Alam 40100, Malaysia; niuhaixu@haut.edu.cn; 2 School of Management, Henan University of Technology, Zhengzhou 450001, China; yhli@haut.edu.cn; 3 College of Information Science and Engineering, Henan University of Technology, Zhengzhou 450001, China; zhangchunyu@stu.haut.edu.cn (C.Z.); ljsun@haut.edu.cn (L.S.)

**Keywords:** wireless sensor network, coverage control, bald eagle search algorithm, Lévy flight, quasi-reflection-based learning, quadratic interpolation, orthogonal learning

## Abstract

Coverage control is a fundamental and critical issue in plentiful wireless sensor network (WSN) applications. Aiming at the high-dimensional optimization problem of sensor node deployment and the complexity of the monitoring area, an orthogonal learning multi-strategy bald eagle search (OLMBES) algorithm is proposed to optimize the location deployment of sensor nodes. This paper incorporates three kinds of strategies into the bald eagle search (BES) algorithm, including Lévy flight, quasi-reflection-based learning, and quadratic interpolation, which enhances the global exploration ability of the algorithm and accelerates the convergence speed. Furthermore, orthogonal learning is integrated into BES to improve the algorithm’s robustness and premature convergence problem. By this way, population search information is fully utilized to generate a more superior position guidance vector, which helps the algorithm jump out of the local optimal solution. Simulation results on CEC2014 benchmark functions reveal that the optimization performance of the proposed approach is better than that of the existing method. On the WSN coverage optimization problem, the proposed method has greater network coverage ratio, node uniformity, and stronger optimization stability when compared to other state-of-the-art algorithms.

## 1. Introduction

Wireless sensor networks (WSNs), as the core foundation of the Internet of Things (IoT) technology [1,2], have garnered immense amounts of attention due to their flexibility, timeliness, scalability, and rapid deployment [3,4]. They are composed of numerous microsensor nodes, which can promptly collect and process real-time data from the monitoring region [5]. In recent years, WSNs have been introduced into intelligent transportation systems [6], military defence [7], environmental monitoring [8,9], medical care [10], and other fields. A highly reliable and robust WSN has created great convenience for human production and life. In multiple sensor network applications, coverage control of the surveillance region is a substantial task that is strongly related to the service quality of WSN. The random deployment of sensor nodes results in inefficient network coverage and an uneven distribution of nodes, affecting the effective collection and transmission of data in the surveillance area. Therefore, it is exceedingly significant to deploy a specific number of sensors to maximize the coverage ratio of the surveillance area.

Immense amounts of specialists and professors have conducted extensive research for the sake of excellent coverage performance [11,12,13]. At present, coverage control algorithms are mainly divided into two categories: centralized and distributed coverage control algorithms. Generally, the distributed approach allows each node to utilize a neighbours’ location information to move repeatedly until it reaches the optimal deployment location. However, in distributed coverage control algorithms, the higher energy consumption in the iterative moving process is inevitable, and thus the network’s total energy consumption decreases significantly. For centralized coverage control algorithms, a sink sensor should be required to perform the coverage optimization with global topology. After analyzing all of the data, it can determine where all of the other sensors in the network should be placed. In comparison with distributed algorithms, centralized coverage optimization algorithms reduce the unnecessary movement of sensors and prolong the network lifetime.

With the development of artificial intelligence theory, a series of swarm intelligence optimization algorithms have emerged, which play an increasingly significant role in optimization problems [14,15,16]. An increasing number of scholars have applied them to the coverage optimization problem of WSN and obtained specific achievements. Multiple swarm intelligence optimization algorithms provide efficient and reliable solutions for solving the optimization problem of sensor node deployment, for instance, particle swarm optimization algorithm (PSO), grey wolf optimization algorithm (GWO), whale optimization algorithm (WOA), invasive weed algorithm (IWO), salp swarm algorithm (SSA), and so forth. The bald eagle search (BES) algorithm [17], proposed by Alsatter in 2020, is a new swarm intelligence optimization algorithm that was inspired by the hunting strategy or intelligent social behaviour of bald eagles. In contrast to other swarm intelligence optimization algorithms, BES has the advantages of high optimization accuracy and fast convergence rate [18], and is widely used in synchronous optimization feature selection [19], support vector machine regression parameter adjustment [20], photovoltaic (PV) model parameter adjustment [21] and other fields. However, BES still suffers from the problem of being easily trapped in local optimums and imbalance between global search and local exploitation. Some scholars have improved the BES algorithm and applied it to the optimization problems. In order to improve the global search ability of the BES algorithm, Zhao et al. [22] incorporated the golden sine algorithm and crisscross strategy into the standard BES algorithm. The improved BES algorithm was applied to the optimization of the back propagation (BP) neural network model, and the experimental results show that the optimized BP neural network model can effectively improve the accuracy of air quality prediction. Ding et al. [23] introduced the adaptive inertia weight and Cauchy mutation strategy into the BES algorithm, which enhanced the local search ability of the algorithm and reduced the possibility of falling into the local optimal. The improved BES shows good optimization ability in engineering applications such as the pressure vessel design. Shen et al. [24] integrated tent chaotic mapping, Lévy flight, and adaptive weights into the BES and applied them to the offloading task of vehicular networks. Simulation results show that the improved BES can effectively reduce the total cost of offloading tasks. Tong et al. [25] introduced a chaos operator and sine and cosine into the BES algorithm and applied them to the position optimization of a logistics distribution centre. Experimental results show that the modified algorithm can effectively save delivery costs and lift efficiency.

For the deployment problem of sensor nodes, most swarm intelligence optimization algorithms still have several shortcomings, such as premature convergence, poor population diversity in the late iterations, and inability to balance the relationship between exploitation and exploration, which will lead to numerous tiny coverage holes and node redundancy. In addition, excessive studies only paid attention to elevating the coverage rate of the region, but limited works have devoted to improving node uniformity while ensuring sufficient coverage. As a novel swarm intelligence optimization algorithm, the applications of BES in WSN are relatively few at present, especially for the deployment problem of sensor nodes. Hence, the motivation that impelled us to conduct further research is to overcome the above drawbacks for the sake of sufficient coverage of the monitoring area and excellent node uniformity. However, BES has a chronic deficiency in terms of weak robustness while dealing with high-dimensional complicated problems, which sometimes results in an inferior solution. Significantly, it is a novel academic idea to enhance the optimization performance of the BES algorithm and apply it to address the problem of sensor node deployment. Therefore, this paper proposes an orthogonal learning multi-strategy bald eagle search (OLMBES) algorithm and applies it to WSN coverage optimization successfully. A series of simulation experiment results show that the proposed method exhibits remarkable performance in the WSN coverage optimization problem, which verifies the effectiveness and superiority of the OLMBES algorithm. The primary contributions of this paper are demonstrated in the following three facets:

(1) A mathematical model for a coverage optimization problem is formulated. To facilitate quantitative analysis, the continuous surveillance area is discretized into multiple target monitoring points.
(2) An OLMBES algorithm is proposed. To begin with, this paper incorporates three kinds of strategies into the BES algorithm, including Lévy flight, quasi-reflection-based learning, and quadratic interpolation, which enhances the global exploration ability of the algorithm and accelerates the convergence speed. Furthermore, orthogonal learning is integrated into the BES algorithm in order to prevent the algorithm trapping in the local optima and to strengthen the robustness of the proposed method.
(3) The performance of the OLMBES algorithm is verified on CEC2014 benchmark functions, applying it to the coverage optimization problem of the WSN. With a series of comparative experimental simulation results, the OLMBES algorithm is confirmed as the most excellent method to tackle with coverage optimization of the WSN compared with the state-of-the-art methods, exhibiting a remarkable performance in terms of coverage rate and node uniformity.

The remainder of the paper is arranged as follows. Section 2 introduces related works on sensor node deployment methods. Section 3 introduces the probabilistic perception model of sensors and coverage performance evaluation indicators. The hunting process of the standard BES algorithm is briefly sketched in Section 4. Section 5 describes the proposed method in detail. The application of the OLMBES algorithm to coverage optimization of the WSN is presented in Section 6. An analysis of experimental simulations is discussed in Section 7. In the end, conclusions and future improvements are presented in Section 8.

## 2. Related Works

In deployment, according to the function of a network, coverage is the most important performance metric for a WSN, and it expresses the ability of the network to monitor an area of interest, meaning that all points within this area are always monitored. To ensure the service quality of a WSN, the coverage optimization problem as a basic research task should be brought to the forefront of public attention.

At present, coverage optimization algorithms are mainly classified as either distributed or centralized. For the distributed coverage optimization methods, each sensor node determines its position at each timestep based on the local information it has received from neighbouring nodes, and the distributed coverage control algorithms can be divided into two groups: force-based and geometrical algorithms [11]. The coverage control algorithms proposed under the forced-based group are inspired by natural phenomena, such as animal aggregation [26] or the equilibrium of molecules [27]. In the force-based group, sensor nodes move based on the force entered from their neighbouring nodes to distribute uniformly in the area, and every sensor node calculates the entered force based on information obtained from neighbouring nodes. In the second group of geometrical algorithms, the Voronoi diagram is the most commonly used structure in WSN [28]. A Voronoi diagram splits the region of interest into cells, and every sensor node undertakes a cell to cover [29].

In a centralized coverage optimization algorithm, the placement of a sensor node is decided by a centralized sensor which is usually called a sink. The sink sensor analyses all of the data and determines where all of the other sensors in the network should be placed. A major problem in deploying sensor nodes is that their area coverage should be maximized. In recent years, numerous experts and scholars have noticed the potential of swarm intelligence optimization algorithms that use nature-inspired computational methodologies in solving high-dimensional complex problems. Several swarm intelligence optimization algorithms have been applied to cope with the sensor deployment problem. Zhao et al. [30] integrated chaotic optimization methods into PSO for the purpose of better coverage performance, which increased the coverage ratio of a monitoring region to a certain extent and improved the phenomenon of the uneven distribution of nodes. Miao et al. [31] proposed an improved GWO with an enhanced hierarchical structure, enhancing the global search ability of the GWO with a new position update equation of grey wolf individuals. Moreover, the proposed approach was found to be usable and efficient in solving the WSN coverage optimization problem, reducing the blind area in the monitoring zone. To improve coverage optimization performance, Wang et al. [32] added the notion of reverse learning to a standard WOA, which improved the node utilization rate while increasing the coverage ratio. Zhu et al. [33] presented a hybrid algorithm of IWO and a differential evolution (DE) algorithm, integrating Lévy flight and random walk strategy into the hybrid algorithm to improve the coverage redundancy and insufficient coverage caused by an uneven distribution of nodes in the surveillance zone. To a degree, it avoided falling into the local optimal solution, and its convergence speed was accelerated. Considering the energy consumption of sensors, Zhang et al. [34] analyzed the relationship between redeployment positions optimized by the DE algorithm and the initial positions of nodes, which effectively reduced the average moving distance and energy consumption of nodes while maintaining a high coverage rate. Bat algorithm (BA), inspired by the foraging behaviour of bats’ echolocation, is employed in WSN coverage optimization due to its rapid convergence and ease of implementation. Mohar et al. [35] introduced an improved BA to optimize node deployment. Nevertheless, this proposed method failed to demonstrate strong robustness due to its many parameter settings, influencing coverage performance greatly. Wang et al. [36] adopted a water wave optimization (WWO) algorithm for location deployment optimization of sensor nodes since it has the advantages of easy operation, fewer control parameters, and powerful search ability. Li et al. [37] proposed a node deployment method based on autonomous multi-decision PSO to improve the coverage ratio of the WSN. Chaotic mapping, multi-decision learning, Cauchy mutation, and reverse learning strategies are integrated into the PSO algorithm to enhance the optimization ability in high-dimensional optimization problems. Zhao et al. [38] proposed an improved ant lion optimization algorithm to optimize the sensor node deployment problem. This algorithm employed the cuckoo search (CS) algorithm and Cauchy mutation strategy to update the positions of ants in the population. The DE algorithm is used to update the position of the ant lion population. On the one hand, this method improves the network coverage performance and reduces the cost of node deployment. On the other hand, the hybrid optimization algorithm greatly increases computational complexity. To improve the network coverage performance, Dao et al. [39] divided the monitoring area into multiple sub-regions, integrated the two strategies of reverse learning and multi-directional technology into the Archimedes optimization algorithm (AOA), and then combined the optimal node locations searched in the sub-regions to obtain an optimal deployment scheme.

These above mentioned optimization methods have improved the coverage ratio of the monitoring region to some extent, but there are still some common drawbacks in solving the problem of sensor deployment. The primary shortcomings include an inefficient coverage rate, coverage redundancy, and poor node uniformity. Most studies focus on coverage rate maximization, ignoring the influence of sensor node uniformity on WSN, which will lead to a redundant coverage, excessive energy consumption of nodes, and thus affect service quality of WSN. Aside from that, BES is a novel swarm intelligence optimization algorithm proposed in recent years, and it has the advantages of high optimization accuracy and fast convergence rate. However, the coverage maximization problem is considered a high-dimensional complex problem, and the high dimensionality directly affects the optimization performance of the BES algorithm. Based on the previous research, considering the influence of node uniformity comprehensively, a new sensor deployment method using the improved BES algorithm is proposed to improve the coverage performance of WSN.

## 3. WSN Coverage Optimization Problem

In the two-dimensional surveillance area with length *L* and width *W*, a set of mobile sensors *S* = {*s*_1_, *s*_2_, *s*_3_, ⋯, *s_N_*} are randomly deployed on the surface of the region, where the position of the *ith* sensor is represented as *s_i_* = {*x_i_*, *y_i_*}, *i* = {1, 2, 3, ⋯, *N*}. Mobile sensors are moved to the optimal position by conducting the specific coverage control algorithm, achieving the maximal coverage ratio of the observation region. Make the following assumptions in this paper:

(a) All sensors are identical in terms of structure, computational power, communication power, storage energy, and synchronous clock.
(b) Each sensor can acquire location information about its own and neighbouring nodes.
(c) The communication radius *R_c_* of each sensor is twice the range of the sensing radius *R_s_*.
(d) In the observation region, there are no obstacles. Each mobile sensor has enough power to perform position update.

**Figure 1:**
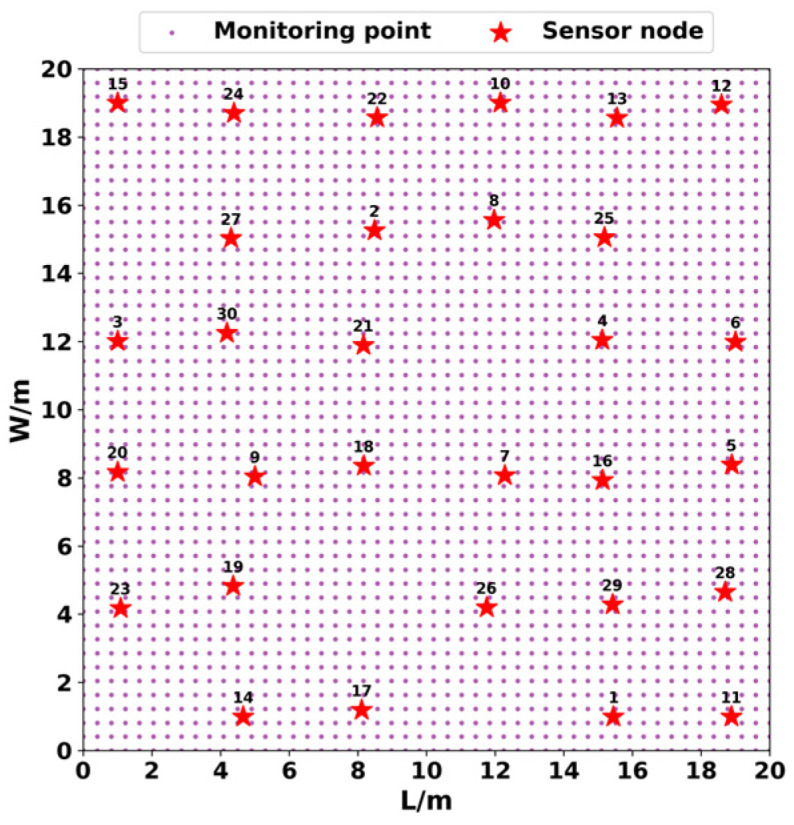
Discrete monitoring area.

**Figure 2:**
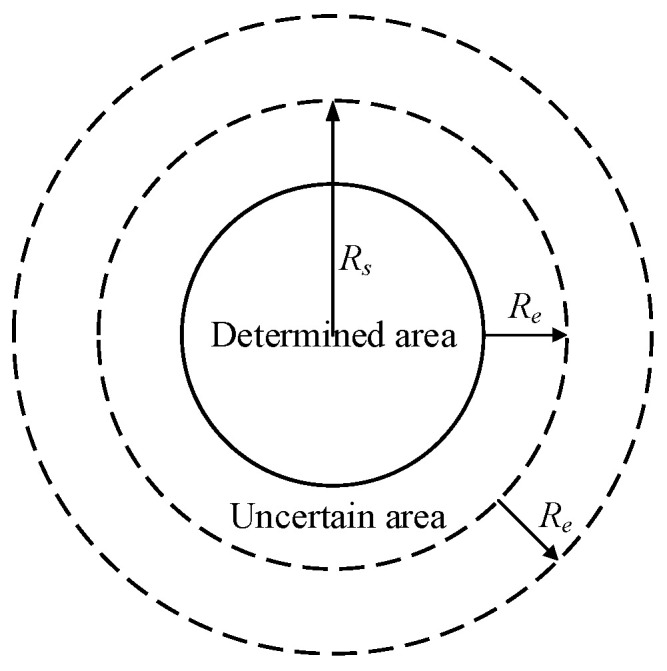
Probabilistic sensing model.

The surveillance region is discretized into *a* × *b* grid points to evaluate the coverage performance indicators effectively, where the *jth* target point in the region is represented as *o_j_* = {*x_j_*, *y_j_*}, *j =* 1, …, *a* × *b*. The smaller the distance between target points, the higher the accuracy of the coverage ratio. Figure 1 shows the discretization process of the monitoring area. This paper adopts the probabilistic perception model since it can simulate the information monitoring process in the actual deployment environment. Figure 2 displays a schematic diagram of the probabilistic perception model.

The distance between sensor *s_i_* and grid point *o_j_* is expressed as

(1) $$d\left(s_{i},o_{j}\right)=\sqrt{{\left(x_{i}-x_{j}\right)}^{2}+{\left(y_{i}-y_{j}\right)}^{2}}$$

The perception probability of node *s_i_* to grid point *o_j_* is defined by

(2) $$\rho \left(s_{i},o_{j}\right)=\left\{\begin{array}{ll} 0 & d\left(s_{i},o_{j}\right)>R_{s}+R_{e}\\ \exp\left(\frac{-\alpha_{1}\lambda_{1}^{\beta_{1}}}{\lambda_{2}^{\beta_{2}}+\alpha_{2}}\right) & R_{s}-R_{e}\leq d\left(s_{i},o_{j}\right)\leq R_{s}+R_{e}\\ 1 & d\left(s_{i},o_{j}\right)<R_{s}-R_{e} \end{array}\right.$$

where *R_e_* is the perceived reliability parameter of sensors, and *α*_1_, *α*_2_, *β*_1_, *β*_2_ are the corresponding parameters related to the property of sensors. In general, *α*_1_ = 1, *α*_2_ = 0, *β*_1_ = 1, *β*_2_ = 1. *λ*_1_, and *λ*_2_ are defined as

(3) $$\left\{\begin{array}{c} \lambda_{1}=R_{e}-R_{s}+d\left(s_{i},o_{j}\right)\\ \lambda_{2}=R_{e}+R_{s}-d\left(s_{i},o_{j}\right) \end{array}\right.$$

Therefore, the joint sensing probability of multiple sensors in the monitoring area to grid point *o_j_* is expressed as

(4) $$\rho_{\mathrm{cov}}\left(S,o_{j}\right)=1-\prod_{i=1}^{N}\left(1-\rho \left(s_{i},o_{j}\right)\right)$$

## 4. Bald Eagle Search Algorithm

The bald eagle is a kind of huge raptor belonging to the Accipitridae family, which mainly feeds on large fish and small mammals that dwell near water [40]. They have acute vision as well as outstanding flight ability, allowing them to quickly locate and swoop to catch their preys. In the process of foraging, bald eagles identify and choose a search space with more preys according to self-searching or tracking the population, flying towards a specific area. Once a target prey is determined, bald eagles will promptly swoop to catch the prey. The BES algorithm mimics the behaviour of bald eagles during predation. Correspondingly, this algorithm is divided into three stages, namely, selecting the search space, searching within the selected area, and swooping [41].

(1) Select stage

To determine an optimal hunting area, bald eagles select a search space with plentiful preys and fly spirally within the selected area. Position update equation of bald eagles in determining the search space stage is indicated using Equation (5)

(5) $$Q_{i}^{new}=Q^{*}+\delta \cdot r\cdot \left(Q^{mean}-Q_{i}\right)$$

where *Q_i_* indicates the position of the *ith* bald eagle individual, Q* and *Q^mean^*, respectively, represent the optimal search position and mean position obtained in the previous probe of population, parameter *δ* influences the variations in position that takes a value between 1.5 and 2, and *r* is a random number that ranges from 0 to 1.

(2) Search stage

After identifying the optimal search space, bald eagles spirally fly to expedite the speed of search in the specific area with might and main. They search for preys around the *Q^mean^* (mean position of population), moving in spiral direction. We use the polar coordinate equation to describe the process of position update, as shown in Equations (6)–(9):

(6) $$Q_{i}^{new}=Q_{i}+u\left(i\right)\cdot \left(Q_{i}-Q^{mean}\right)+v\left(i\right)\cdot \left(Q_{i}-Q_{i+1}\right)$$

(7) $$u\left(i\right)=\frac{ur\left(i\right)}{\max\left(\left|ur\right|\right)},v\left(i\right)=\frac{vr\left(i\right)}{\max\left(\left|vr\right|\right)}$$

(8) $$ur\left(i\right)=r\left(i\right)\cdot \sin\left(\theta \left(i\right)\right),vr\left(i\right)=r\left(i\right)\cdot \cos\left(\theta \left(i\right)\right)$$

(9) $$\theta \left(i\right)=\alpha \cdot \pi \cdot rand,r\left(i\right)=\theta \left(i\right)+R\cdot rand$$

where *α* is a parameter that controls the angle between adjacent search points, taking a value between 5 and 10, and *R* is used to determine the number of search cycles that takes a value from 0.5 to 2.

(3) Swooping stage

In the swooping stage, the bald eagles descend from the optimal subduction position to capture the target prey. In the meanwhile, other individuals in the population also move promptly to the optimal position and attack preys. Likewise, this paper also adopts the polar coordinate equation to describe the position update of the swooping stage, as shown in Equations (10)–(13):

(10) $$Q_{i}^{new}=rand\cdot Q^{*}+ul\left(i\right)\cdot \left(Q_{i}-c_{1}\cdot Q^{mean}\right)+vl\left(i\right)\cdot \left(Q_{i}-c_{2}\cdot Q^{*}\right)$$

(11) $$ul\left(i\right)=\frac{ur\left(i\right)}{\max\left(\left|ur\right|\right)},vl\left(i\right)=\frac{vr\left(i\right)}{\max\left(\left|vr\right|\right)}$$

(12) $$ur\left(i\right)=r\left(i\right)\cdot \sinh\left(\theta \left(i\right)\right),vr\left(i\right)=r\left(i\right)\cdot \cosh\left(\theta \left(i\right)\right)$$

(13) $$\theta \left(i\right)=\alpha \cdot \pi \cdot rand,r\left(i\right)=\theta \left(i\right)$$

where *c*_1_, *c*_2_ ∈ [1, 2].

**Figure 3:**
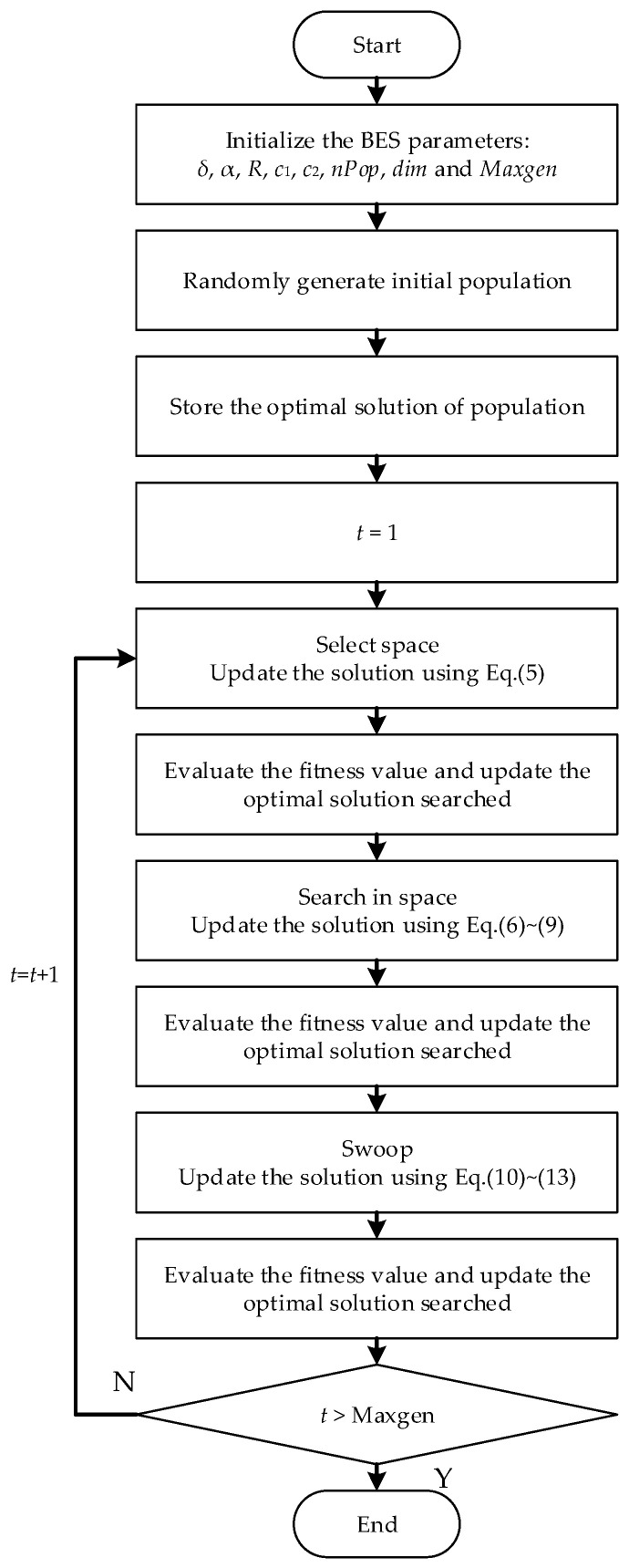
Flowchart of the BES algorithm.

A flowchart depicting the BES algorithm is exhibited in Figure 3.

## 5. Proposed Methodology

### 5.1. Lévy Flight

**Figure 4:**
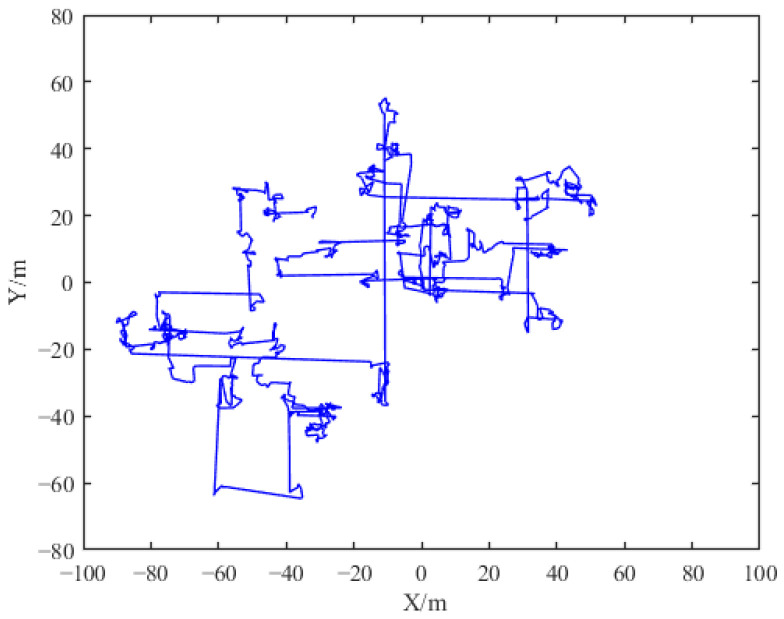
Trajectory diagram of *Lévy* flight.

The standard BES algorithm heavily relies on search information in the stage of selecting the search space. It is inefficient to merely search for new space near the global optimal solution, which leads to a sluggish convergence speed and stagnation in the local optimal solution. Therefore, the BES algorithm cannot exhibit remarkable optimization performance when optimizing complicated practical problems. *Lévy* flight, as a kind of random walk, has a paramount characteristic of executing occasional leaps interspersed with several tiny steps, which helps the population to seek a more potential search space and jump out of the local optimal solution. The position update equation is indicated in Equation (14)

(14) $$Q_{i}^{levy}=Q^{*}+sign\left(rand-\frac{1}{2}\right)\cdot Levy\left(\lambda \right)$$

where *rand* is a random number in the interval [0, 1] obeying uniform distribution, *sign*() is expressed as a sign function, and *Lévy*(*λ*) represents the route that obeys *Lévy* distribution. The calculation equations for *Lévy* flight are as follows

(15) $$Levy\left(\lambda \right)=\frac{\mu \cdot \sigma }{{\left|\nu \right|}^{1/\lambda }}$$

(16) $$\mu \sim N\left(0,\sigma_{\mu }^{2}\right),\nu \sim N\left(0,\sigma_{\nu }^{2}\right),\sigma_{\mu }=\sigma_{\nu }=1$$

(17) $$\sigma ={\left[\frac{\Gamma \left(1+\lambda \right)\cdot \sin\left(\pi \lambda /2\right)}{\Gamma \left(\left(1+\lambda \right)/2\right)\cdot \lambda \cdot 2^{\left(\lambda -1\right)/2}}\right]}^{1/\lambda }$$

where *μ* and *ν* obey the standard normal distribution, *σ_μ_* = *σ_ν_* = 1, their dimensions are consistent with each individual in the population, and *λ* is generally taken as 1.5. Figure 4 presents a simulated image of the *Lévy* flight path.

### 5.2. Quasi-Reflection-Based Learning

Bald eagles spirally fly in the selected region to search for prey. It is distinctly possible to miss a more remarkable solution due to inefficient exploration within the selected space. The primary concept of quasi-reflection-based learning (QRBL) is to calculate and evaluate the current solution vector and quasi-reflection solution at the same time, and then choose the solution equipped with better fitness to enter the next iteration [42]. This method can effectively raise the population diversity and speed up convergence.

**Figure 5:**
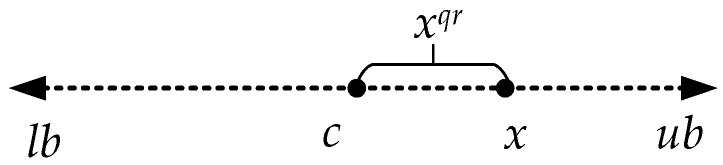
Quasi-reflection points defined in domain [*lb*, *ub*].

If *x* is a point in the search interval [*lb*, *ub*] and *c* = (*lb* + *ub*)/2 represents the midpoint of the search interval, then the quasi-reflection point corresponding to point *x* can be calculated using Equation (18). The relative positional relationship between a random point and its quasi-reflection point in the search interval is depicted in Figure 5.

(18) $$x^{qr}=rand\left(c,x\right)$$

In a high-dimensional vector space, the quasi-reflection mechanism can be applied to each dimension, as shown in Equation (19):

(19) $$Q_{i,j}^{qr}=rand\left(\frac{lb_{j}+ub_{j}}{2},q_{i,j}\right)$$

### 5.3. Quadratic Interpolation

In the swooping stage, bald eagles swoop rapidly to capture a target prey. In the meanwhile, other individuals also move to the optimal position, which leads to inferior population diversity and stagnation in the local optimal solution. Quadratic interpolation (QI), as a type of nonlinear crossover operator, approximately fits the shape of quadratic curve through three solution vectors in the population, generating a new solution vector by mutation [43]. In this paper, QI is applied to position update strategy of a random individual in the population, guiding by the top three solution vectors. Position update equation is indicated in Equation (20):

(20) $$Q^{mu}=\frac{1}{2}\frac{\left[{\left(Q^{t}\right)}^{2}-{\left(Q^{s}\right)}^{2}\right]\cdot f\left(Q^{*}\right)+\left[{\left(Q^{*}\right)}^{2}-{\left(Q^{t}\right)}^{2}\right]\cdot f\left(Q^{s}\right)+\left[{\left(Q^{s}\right)}^{2}-{\left(Q^{*}\right)}^{2}\right]\cdot f\left(Q^{t}\right)}{\left(Q^{t}-Q^{s}\right)\cdot f\left(Q^{*}\right)+\left(Q^{*}-Q^{t}\right)\cdot f\left(Q^{s}\right)+\left(Q^{s}-Q^{*}\right)\cdot f\left(Q^{t}\right)}$$

where Q*, *Q^s^,* and *Q^t^,* respectively, represent the top three solution vectors of fitness, and *f*(.) represents its fitness value.

### 5.4. Orthogonal Learning Strategy

(1) Orthogonal experimental design

Orthogonal experimental design (OED) is an experimental design method used to study multi-factor and multi-level problems, using the least number of experiments to achieve equivalent results with comprehensive experiments [44]. Considering an optimization problem with fitness related to *Z* factors, each factor is assigned to one of *H* levels. If the experimenter adopts the exhaustive method to calculate all test combinations, we are supposed to evaluate *H^Z^* calculations to seek the optimal solution. When the value of *H* or *Z* is large, it is time-consuming and inefficient to find the best combination.

Orthogonal table (OA) is an extraordinarily crucial tool in OED. According to the orthogonality of the orthogonal table, representative test combinations can be selected from comprehensive experiments to reduce the amount of calculation [45]. *L_M_*(*H^Z^*) signifies an orthogonal array with *Z* factors and *H* levels per factor, where *L* is the orthogonal array and *M* denotes the number of test combinations. An orthogonal table with four factors and three levels per factor is expressed in Equation (21). Assuming an optimization problem has four factors and three levels, 3^4^ = 81 experimental calculations are required to seek the optimal combination if we adopt the exhaustive method. Nevertheless, we only calculate 9 experimental results using the OED method.

(21) $$L_{9}\left(3^{4}\right)=\left[\begin{array}{cccc} 1 & 1 & 1 & 1\\ 1 & 2 & 2 & 2\\ 1 & 3 & 3 & 3\\ 2 & 1 & 2 & 3\\ 2 & 2 & 3 & 1\\ 2 & 3 & 1 & 2\\ 3 & 1 & 3 & 2\\ 3 & 2 & 1 & 3\\ 3 & 3 & 2 & 1 \end{array}\right]$$

Factor analysis (FA) can judge the influence of each level on each factor according to the fitness value of *M* test combinations [46]. *f_m_* represents fitness value of the *mth* test combination (*m* = 1, 2, 3, …, *M*). *S_zh_* indicates the impact degree of the *hth* level (*h* = 1, 2, …, *H*) on the *zth* factor (*z* = 1, 2, …, *Z*); the calculation process is expressed in Equation (22).

(22) $$S_{zh}=\frac{\sum_{m=1}^{M}f_{m}\times e_{mzh}}{\sum_{m=1}^{M}e_{mzh}}$$

where *e_mzh_* is 1 if the level of the *zth* (*z* = 1, 2, ⋯, *Z*) factor of the *mth* (*m* = 1, 2, ⋯, *M*) test combination is *h* (*h* = 1, 2, ⋯, *H*). Otherwise, *e_mzh_* is 0. For an optimization problem, the larger the *S_zh_* is, the better the *hth* level on factor *z* will be. Otherwise, vice versa.

(2) Orthogonal learning strategy

In the BES algorithm, the optimal position of population plays an indispensable role in guiding other individuals to hunt for preys. To further enhance the global search ability, the orthogonal learning strategy is integrated into the BES algorithm, fully utilizing the search information of the population to help it find a better position guiding vector and avoid falling into the local optimal solution.

For high-dimensional optimization problems, there are several preparatory works to conduct before embedding the orthogonal learning strategy into the algorithm. First of all, the solution vector needs to be divided into *k* groups so as to reduce the number of factors, with each group corresponding to a factor. Furthermore, it is necessary to construct several levels for each factor so that comprehensive information can be obtained from each factor. This paper constructs four levels for each factor, and the construction process is given as follows:

(a) The global optimal solution vector Q* with the best fitness value is chosen.
(b) The second optimal solution vector *Q^s^* with suboptimal fitness value is selected.
(c) A random solution vector *Q_i_* that differs from Q* and *Q^s^* is determined.
(d) The centroid opposition-based solution vector $\bar{Q_{i}}$ corresponding to *Q_i_* is calculated. The calculation process of $\bar{Q_{i}}$ is given as follows:

(23) $$\bar{Q_{i}}=2G-Q_{i}$$

(24) $$G=\frac{Q_{1}+Q_{2}+\cdots +Q_{nPop}}{nPop}$$

where *G* represents the gravity centre of the population. The search space of reverse points of the gravity centre is a dynamic boundary, denoted as *q_ij_* ∈ [*pa_j_*, *pb_j_*]. If the reverse points of the gravity centre surpass the boundary, Equation (26) is used to amend the position of the points.

(25) $$pa_{j}=\min\left(q_{ij}\right),pb_{j}=\max\left(q_{ij}\right)$$

(26) $$\bar{q_{i,j}}=\left\{\begin{array}{c} \begin{array}{cc} pa_{j}+rand\left(0,1\right)\cdot \left(G_{j}-pa_{j}\right) & if\;\;\bar{q_{i,j}}<pa_{j} \end{array}\\ \begin{array}{cc} G_{j}+rand\left(0,1\right)\cdot \left(pb_{j}-G_{j}\right) & if\;\;\bar{q_{i,j}}>pb_{j} \end{array} \end{array}\right.$$

To summarize, four different levels of each factor can be obtained, denoted as *T* = {Q*, *Q^s^*, *Q_i_*, $\bar{Q_{i}}$}. The set of *M* different search solution vectors can be obtained by OED, denoted as *C* = {*C*_1_, *C*_2_, …, *C_M_*}. According to factor analysis, the best combination of different levels of each factor is obtained, generating a new guidance vector *Q^gv^* of the population. The orthogonal learning strategy is indicated by

(27) $$Q^{gv}=OED\left(Q^{*},Q^{s},Q_{i},\bar{Q_{i}}\right)$$

The orthogonal learning strategy (OLS) helps the population to jump out of the local optimal solution and speeds up convergence. When the fitness value of the optimal solution falls into stagnation, the OLS can help it find a new guidance vector that potentially facilitates a more remarkable solution. However, overuse of the OLS may also disrupt original search patterns of bald eagles. Therefore, this paper sets up a triggering mechanism of the OLS, defining a stagnation number parameter, *stagnated_num*. If and only if *stagnated_num* is greater than or equal to *limit* (maximal stagnation times), execute the OLS and then reset *stagnated_num* to 0.

### 5.5. Complexity Analysis of Proposed OLMBES Algorithm

Different algorithms take varying amounts of time to optimize the same problems, and assessing the computational complexity of an algorithm is an essential way to evaluate its execution time. For the proposed OLMBES algorithm, we utilize Big O notation [47] to analyze the time complexity. Let *nPop* represent the population size of the proposed algorithm, and *Maxgen* be the maximum number of iterations. In the OLS, *M* denotes the number of experiments generated by the OLS. Following the symbol *O* rules of operation for the time complexity, the time complexity for randomly initializing the population is *O*(*nPop*). During the solution update process, the computational complexity for the selecting stage, the searching stage and the swooping stage are same as *O*(nPop*Maxgen), which encompasses both finding the best positions and updating the positions of all solutions, and *O*(M*Maxgen) represents the computational complexity of the OLS. Therefore, the total computational complexity of the proposed OLMBES algorithm can be expressed as *O*(Maxgen*(*nPop*+*M*)+*nPop*). Table 1 shows the pseudo-code of the proposed OLMBES algorithm.

## 6. Proposed OLMBES Algorithm for WSN Coverage Optimization

**Figure 6:**
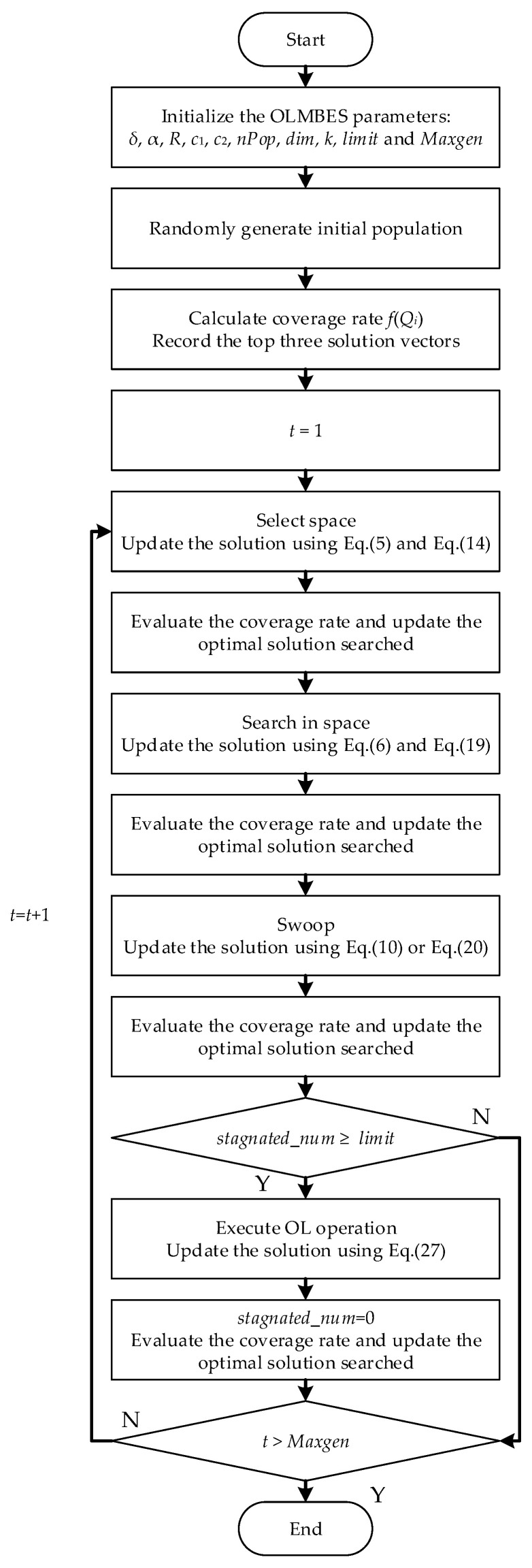
Flowchart of the OLMBES algorithm.

Application of the OLMBES algorithm in WSN coverage optimization is indicated in Figure 6. The procedures of the OLMBES algorithm are depicted below.

Step 1: Initialize the lower bound (*lb*) and upper bound (*ub*) of the monitoring region. At the same time, determine the number of sensor nodes (*N*), denoted as *S* = {*s*_1_, *s*_2_, *s*_3_, ⋯, *s_N_*}.

Step 2: Initialize the parameters of the OLMBES algorithm.

Step 3: Randomly generate positions of *nPop* bald eagles, indicated as *Q* = {*Q*_1_, *Q*_2_, …, *Q_nPop_*}, *Q_i_* = {*q*_1_, *q*_2_, …, *q*_2N−1_, *q*_2N_} (*i* = 1, 2, …, *nPop*). Evaluate the coverage ratio (*f*(*Q_i_*)) of each position set and record the top three fitness values and its corresponding position vectors.

Step 4: Enter the iterative loop. In the stage of selecting the search space, update positions using Equations (5) and (14). Evaluate the coverage ratio of the updated positions and judge whether the updated position is a better choice.

Step 5: In the stage of searching for preys, update positions using Equation (6) and calculate the quasi-reflection position vectors corresponding to the updated positions utilizing Equation (19). Evaluate the coverage ratio of the updated positions and corresponding quasi-reflection position vectors, and then choose the position with greater coverage ratio.

Step 6: In the stage of swooping, randomly choose an individual of the population, using Equation (20) to update the position. In the meanwhile, update the position according to Equation (10) for other individuals. Evaluate the coverage ratio of the updated positions and judge whether the updated position is a better choice.

Step 7: Update the top three position vectors, namely, Q*, *Q^s^*, *Q^t^*. Check whether *stagnated_num* is greater than or equal to *limit*. If *stagnated_num* reaches the stagnation threshold, execute the OLS according to Equation (27), and then reset the *stagnated_num* to 0. Calculate the coverage ratio of the updated position and determine whether the updated position is a better choice.

Step 8: Check whether the number of iterations is greater than *Maxgen*. If not, then *t* = *t* + 1, so go to Step 4. Otherwise, end the iterative loop and output the optimal deployment positions of sensor nodes.

## 7. Simulation Experiments and Analysis

### 7.1. CEC2014 Benchmark Functions Test

**Figure 7:**
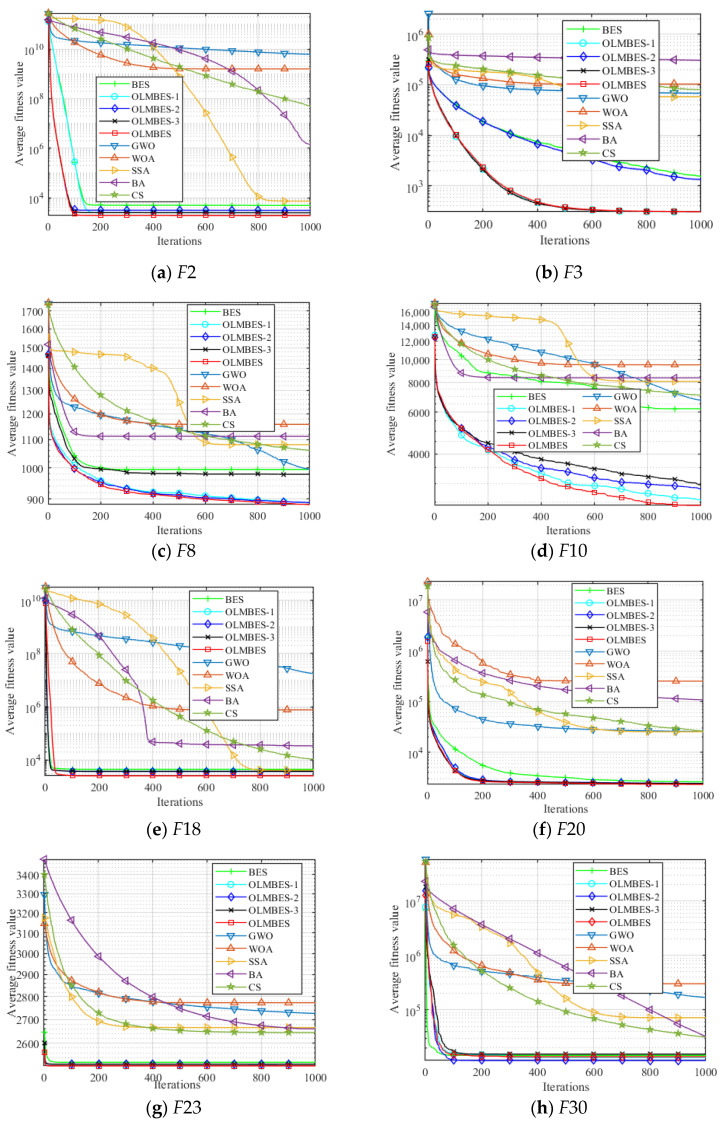
Convergence curves of different algorithms under several benchmark functions.

To verify the effectiveness of the OLMBES algorithm and validate the performance of different strategies embedded in the OLMBES algorithm in solving high-dimensional optimization problems, the CEC2014 benchmark functions set is used to test the performance of the existing algorithms and the proposed OLMBES algorithm. *F*1~*F*3 are unimodal rotation functions. *F*4~*F*16 are simple multimodal functions with shift and rotation. *F*17~*F*22 are hybrid functions. *F*23~*F*30 are composite functions. On 30 test functions of CEC2014, the OLMBES algorithm is compared with the BES, GWO, WOA, SSA, BA, and CS algorithms. In order to justify the effect of different strategies proposed in this paper on the performance improvement of the OLMBES algorithm, the following definitions are made. The OLMBES algorithm not fused with Lévy flight strategy is named OLMBES-1. The OLMBES algorithm without quasi-reflection learning and quadratic interpolation strategies is named OLMBES-2. The OLMBES algorithm without orthogonal learning is named OLMBES-3. The algorithm parameters are set as follows: the population size is 100, dimension is 50, and maximum number of iterations is 1000. Table 2 exhibits the comparison results between the proposed algorithm and the existing algorithms in terms of mean and standard deviation obtained after 30 independent runs of selected CEC2014 test functions. Figure 7 displays the convergence curve of the algorithm on several benchmark functions.

Observing the average fitness values of the OLMBES, BES, GWO, WOA, SSA, BA, and CS algorithms in Table 2 on the CEC2014 test functions set, the algorithm proposed in this paper achieves the best performance on the benchmark functions *F*1~*F*4, *F*7~*F*27 and *F*30, equipping it with the ability to seek solutions closer to the theoretical global optimal value. The SSA, GWO, CS, and BA algorithms show the best average convergence precision on functions *F*5, *F*6, *F*28, and *F*29, respectively. On most benchmark functions, the excellent performance of the OLMBES algorithm on the average fitness value verifies that the proposed algorithm has a strong global search ability compared with the six other algorithms, which can effectively balance the relationship between exploitation and exploration and find a solution closer to the theoretical global optimal value. Observing the standard deviation of the seven algorithms in Table 2 on the test functions, it can be found that the proposed algorithm has the smallest standard deviation value on the *F*1~*F*3, *F*8, *F*11~*F*13, *F*15, *F*17~*F*25, and *F*30 functions. The stability of the OLMBES algorithm is the best among the seven algorithms. Among the six other algorithms, the CS algorithm has the best optimization stability on the *F*4, *F*6, *F*9~*F*10, *F*14, *F*16, *F*26, and *F*28~*F*29 functions, and its stability ranks second among the seven algorithms. BA has the most stable optimization ability on the *F*5 and *F*26 functions. SSA has the best standard deviation on function *F*7. On the CEC2014 benchmark functions, the OLMBES algorithm has the strongest optimization ability and optimization stability compared with the other six algorithms, which provides a new research direction for solving high-dimensional complex problems.

By observing the mean value of the OLMBES-1, OLMBES-2, OLMBES-3, and OLMBES algorithms in Table 2, it is found that the average fitness values of OLMBES-1, OLMBES-2, OLMBS-3, and OLMBES algorithms are smaller than the BES and the other five algorithms on most benchmark functions, and the OLMBES algorithm has the best performance on the mean indicator except *F*22, *F*28, and *F*30. This proves that Lévy flight, quasi-reflection learning, quadratic interpolation, and orthogonal learning strategies can enhance the convergence precision of the algorithm. On the most benchmark functions, the OLMBES-1 algorithm performs better than the BES, OLMBES-2, and OLMBES-3 algorithms on the mean values except *F*5, *F*6, *F*8, and *F*30. The OLMBES-2 algorithm has better performance than the BES and OLMBES-3 algorithms on mean value. Except for the *F*6, *F*17, *F*22, *F*26~*F*28, and *F*30 functions, the OLMBES-3 algorithm has better optimization ability than the BES algorithm. From the perspective of improving the convergence precision of the algorithm, the orthogonal learning strategy has the greatest impact on the algorithm, which can effectively prevent falling into the local optimal solution. However, the influence of quasi-reflection learning and quadratic interpolation strategies are greater than Lévy flight. By observing the standard deviation index of these algorithms in Table 2, the OLMBES and its variant algorithms have superior robustness to other algorithms in the optimization of most benchmark functions. In general, the four strategies embedded in the OLMBES algorithm make the optimization performance more stable.

### 7.2. Simulation Experiments on WSN Coverage Optimization

#### 7.2.1. Comparison of Coverage Performance

To verify the superiority and effectiveness of the proposed OLMBES algorithm in a WSN coverage optimization problem, the BES, GWO, WOA, SSA, BA, and CS algorithms are compared with the proposed method in the same surveillance area. The coverage rate and node uniformity are primary indicators to effectively evaluate the quality of solutions. The fitness function of a coverage optimization task is maximization of the coverage rate. In the meanwhile, the uniformity of sensor nodes is considered a crucial index to judge the quality of the optimal solutions. Make the following definitions:

(1) The coverage rate is one of the indispensable indicators used to evaluate coverage performance [48]. The greater the coverage rate is, the more comprehensive information sensors will collect. Assume that the coverage rate (*CR*) is defined as the ratio of the sum of joint sensing probability for all grid points to the total number of grid points, as shown in Equation (28).

(28) $$CR=\frac{\sum_{j=1}^{a\times b}\rho_{\mathrm{cov}}\left(S,o_{j}\right)}{a\times b}$$

(2) Uniformity is an indicator to measure the distribution of sensors in the surveillance area [49]. The smaller the uniformity is, the more even the sensors will distribute. Suppose that uniformity (*U*) is defined as the mean of the standard deviation of distance between sensors and its neighbouring nodes, as shown by

(29) $$U=\frac{1}{N}\sum_{i=1}^{N}\sqrt{\frac{1}{p_{i}}\sum_{j=1}^{p_{i}}{\left(\frac{1}{p_{i}}\sum_{z=1}^{p_{i}}D_{i,z}-D_{i,j}\right)}^{2}}$$

where *D_i,j_* represents the distance between the *ith* and *jth* sensors, *N* denotes the number of sensor nodes, and *p_i_* denotes the number of neighbouring nodes of the *ith* sensor.

The network simulation environment is set as follows. The size of the monitoring area is 20 m × 20 m, deploying a specific number of sensors in this region, denoted as *N* = 30. The sensing radius of sensors in the deployment region is *R_s_* = 2.5 m, defining the sensing reliability parameter *R_e_* = 0.8 m. The population size is indicated as *nPop* = 30, and the maximum number of iterations (*Maxgen*) is 500. For the sake of effectively calculating the coverage ratio, the distance between grid points is set to 0.4. We repeatedly executed 10 times under the same experimental environment to reduce the influence of experimental randomness. Table 3 shows the parameter settings of the seven comparison algorithms.

Table 4 shows the average coverage ratio and uniformity after optimization by seven different algorithms. What seems beyond dispute from Table 4 is that the OLMBES algorithm has the most extraordinary coverage performance in terms of the average coverage ratio and uniformity. Compared with the other six algorithms, the average coverage ratio of the OLMBES algorithm is improved by 2.25%, 2.69%, 4.16%, 2.54%, 2.31%, and 3.39%, respectively. In the meanwhile, with the OLMBES algorithm, the average uniformity is improved by 0.177, 0.204 0.249, 0.179, 0.178, and 0.234, respectively.

**Figure 8:**
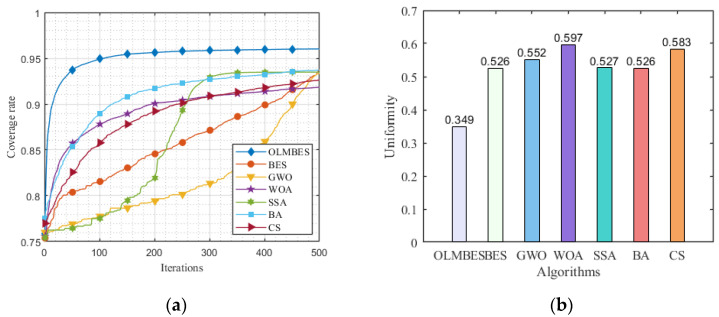
Comparison of coverage performance indicators: (**a**) variation curves of coverage rate changing with iterative times; (**b**) uniformity comparison of algorithms.

Figure 8a shows a graph of the average coverage ratio changing with the number of iterations. It can be seen from Figure 8a that the OLMBES algorithm has the highest average coverage ratio and the fastest convergence speed. In comparison, both the BES and GWO algorithms converge more slowly throughout the iterative process, but their final average coverage rates after optimization are similar and significantly better than that of the WOA and CS algorithms. Additionally, the BA, WOA, and CS algorithms have similar convergence speeds in the early iteration process, but the final coverage rates of the BA is better than that of the WOA and CS algorithms, and comparable to that of the BES and GWO algorithms. Although the SSA has a slower convergence speed during the early iterations, it converges faster in the mid-iteration process, and its final average coverage rate is similar to that of the BA, GWO, and BES algorithms but better than that of the WOA and CS algorithms. Figure 8b shows a histogram of the uniformity of the node distribution in the network after optimization by the seven different algorithms. Since a lower uniformity value indicates a more uniform distribution of sensor nodes, it proves that the OLMBES algorithm has the most remarkable performance on uniformity of sensor nodes.

**Figure 9:**
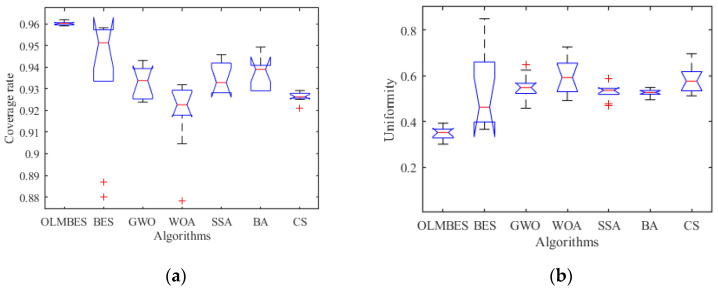
Analysis of algorithm robustness: (**a**) network coverage ratio boxplot diagram; (**b**) uniformity boxplot diagram.

In order to verify the robustness of the OLMBES algorithm, boxplot is employed to describe the data distribution of the coverage ratio and uniformity obtained by running the seven algorithms repeatedly in the same environment, and it is a kind of statistical graph showing the distribution of a set of data by indicating the maximum, minimum, median, upper and lower quartiles, and outliers. Figure 9a shows a boxplot graph of the network coverage rate optimized by the seven algorithms. For the indicator of network coverage rate, a larger value of network coverage rate indicates better coverage performance of sensor nodes in the network. As shown in Figure 9a, the upper quartile, median, and lower quartile of the OLMBES algorithm are higher than the other six algorithms, equipping the shortest interquartile range and no outliers, indicating that the OLMBES algorithm has excellent global search ability and strong robustness. Compared with the other six algorithms, the boxplot height of the OLMBES algorithm is the shortest, which indicates that the OLMBES algorithm has a small degree of data fluctuation and can provide a feasible solution with a high coverage ratio for the coverage optimization problem. In the boxplots of the BES, WOA, and CS algorithms, there are outliers that deviate from the average coverage level, which indicates that the solutions provided by these algorithms in optimizing the deployment of sensor nodes are unstable. Compared with the GWO, WOA, SSA, BA, and CS algorithms, the median line of the BES algorithm is significantly higher than these five algorithms, but the BES algorithm has a longer interquartile range and greater data volatility, further indicating that the BES algorithm has a strong global search ability and poor stability. Similarly, Figure 9b shows a boxplot graph of the uniformity obtained by the seven algorithms. For the network uniformity, a lower value indicates a better distribution performance of sensor nodes in the network. As shown in Figure 9b, the upper quartile, median, and lower quartile of the OLMBES algorithm are all lower than the other six algorithms, indicating that the optimized node deployment position of the OLMBES algorithm has the highest degree of uniformity and robustness.

#### 7.2.2. Influence Comparison of Sensor Nodes Number

To explore the effect of sensor number on the coverage rate and uniformity, the total number of sensors was gradually increased from 24 to 32 (increase two nodes per group) under the same simulation environment. Table 5 shows the performance indicators of the coverage rate when the total number of sensors increases sequentially. It can be seen from Table 5 that the average coverage rate of the seven algorithms elevate with an increase in the total number of sensors when the size of the surveillance area remains unchanged. By contrast, the OLMBES algorithm has the highest coverage rate and the most outstanding uniformity. Table 6 shows the optimized network node uniformity of the seven algorithms for different numbers of nodes. Since a lower value of uniformity indicates a more uniform distribution of network nodes, it can be intuitively seen that the proposed OLMBES algorithm shows the best network node distribution performance for different numbers of nodes, and as the number of nodes increases, the network uniformity improves gradually. With the increase in the number of nodes, the network uniformity values optimized by the CS and WOA algorithms remain around 0.6. In addition, the network uniformity values of the GWO, BA, BES, and SSA algorithms show an increasing trend with the increase in the number of nodes, especially for the GWO algorithm. For the sake of ensuring the higher coverage ratio level of the WSN, the minimum total number of nodes should be 30 for this size of region, according to the theory on the number of theoretical sensors in the literature [50]. Therefore, 30 sensor nodes were used in this paper to maximize the coverage rate for the surveillance area of 20 m × 20 m.

**Figure 10:**
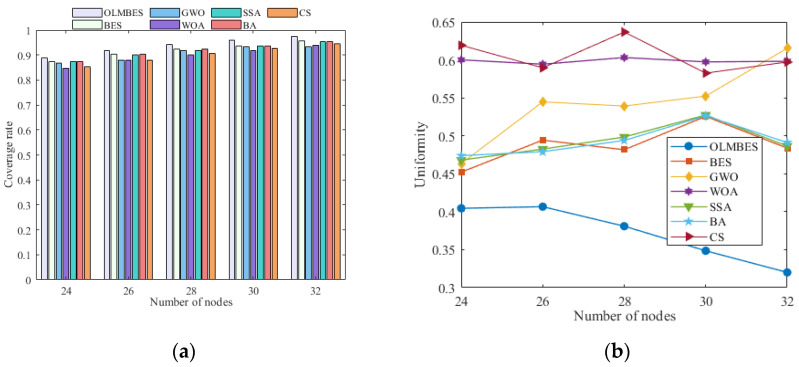
Analysis of the coverage rate and uniformity with the numbers of nodes: (**a**) the average coverage rate histogram for different numbers of nodes; (**b**) the average uniformity line chart for different numbers of nodes.

Figure 10a and Figure 10b, respectively, show a histogram of the average coverage rate and a line chart of uniformity in the same monitoring area with different total numbers of sensor nodes. It can be seen from Figure 10a,b that when the number of sensor nodes is identical, the average coverage rate and uniformity of the solutions provided by the OLMBES algorithm are always better than the other six algorithms.

**Figure 11:**
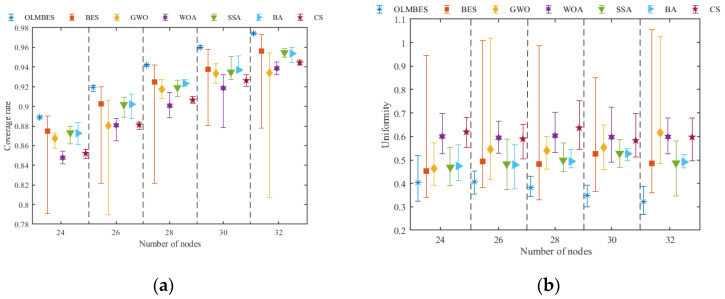
Robustness analysis of the coverage rate and uniformity with the numbers of nodes: (**a**) coverage rate error bar for different numbers of nodes; (**b**) uniformity error bar for different numbers of nodes.

Figure 11a and Figure 11b, respectively, correspond to error bar graphs. By observing Figure 11a, it can be found that for the same number of sensor nodes, the solutions provided by OLMBES algorithm not only have the highest average coverage rate, but also possess the smallest difference between the optimal coverage rate and the worst coverage rate in multiple runs. It is indisputable that the OLMBES algorithm has the strongest stability. The BES algorithm displays distinct instability when optimizing the positions of sensor nodes. The coverage index fluctuates greatly and the stability is the weakest. The node deployment schemes provided by the GWO, WOA, SSA, and CS algorithms cannot meet the requirements for the effective coverage of monitoring areas. Figure 11b shows that the OLMBES algorithm provides the best node deployment scheme in terms of uniformity index compared to the other six algorithms. The proposed method can improve coverage efficiency, ensure the connectivity between nodes, and reduce the occurrence of coverage redundancy.

#### 7.2.3. Effect Comparison of Monitoring Area Size

We set surveillance areas of different sizes and observed the impact of area size on the coverage performance indicators. Table 7 shows the parameter settings for three different surveillance areas, and the rest of the parameter settings remained unchanged. Table 8 and Table 9 show the comparison of the average coverage rates and uniformity in three monitoring areas of different sizes. It can be seen from Table 8 and Table 9 that the OLMBES algorithm can ensure the sufficient coverage of WSN, whatever the size of the surveillance area is.

**Figure 12:**
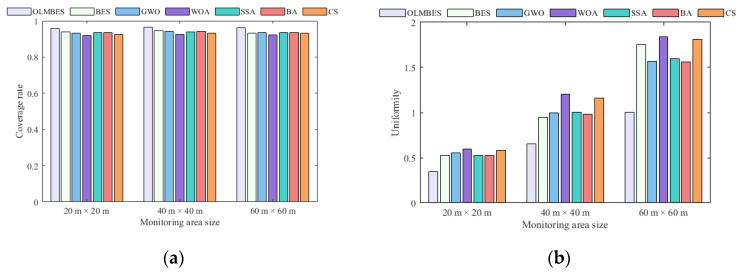
Analysis of coverage rates and uniformity changing with sizes of surveillance area: (**a**) the average coverage rate histogram for different surveillance areas; (**b**) the average uniformity histogram for different sizes of surveillance area.

**Figure 13:**
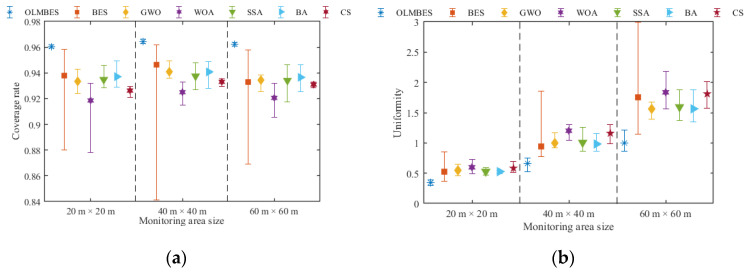
Robustness analysis of the coverage rate and uniformity with different sizes of surveillance areas: (**a**) the coverage rate error bar for different sizes of surveillance areas; (**b**) uniformity error bar for different sizes of surveillance areas.

Figure 12a,b shows histograms of the average coverage rates and uniformity in three monitoring areas of different sizes. Figure 13a and Figure 13b, respectively, correspond to error bar graphs. It can be seen from Figure 12 and Figure 13 that the OLMBES algorithm can ensure the higher regional coverage rate and node uniformity compared with the six other algorithms. The OLMBES algorithm exhibits the most excellent coverage performance and the strongest robustness. By contrast, the other six algorithms have several problems with inefficient coverage rates, poor uniformity of sensor nodes, and unstable optimization performance. To a certain extent, simulation results on different sizes of monitoring areas show that the proposed algorithm has excellent adaptability to the sizes of surveillance regions and can stably provide high-quality node deployment solutions.

### 7.3. Summary and Discussions

In order to comprehensively evaluate the optimization performance of the proposed OLMBES algorithm and its effectiveness in network coverage, we conducted specific simulation experiments on both standard test functions and the application of network coverage control. The compared algorithms in the experiments include the standard BES algorithm as well as several state-of-the-art metaheuristic algorithms, such as GWO, CS, SSA, BA, and WOA.

For the simulation experiments on the standard test functions, we selected the CEC2014 benchmark, which consists of 30 test functions including unimodal functions, multimodal functions, hybrid functions, and composite functions, to comprehensively assess the algorithm optimization performance. First, compared with the other six algorithms, the proposed OLMBES algorithm achieved the lowest average fitness and mean squared error on most benchmark functions, demonstrating its superior optimization accuracy and stability. Then, to further verify the effects of different strategies on optimization performance, the OLMBES algorithm and its variants also displayed excellent performance on most benchmark functions relative to the other six algorithms. Additionally, the convergence curves of some benchmark functions demonstrated the faster convergence speed of the proposed algorithm.

In the network coverage simulation experiments, two evaluation metrics were used: network coverage rate and the uniformity of network node distribution. On this basis, we first conducted experiments under an unchanged network simulation environment and verified that the proposed OLMBES algorithm performed better than the other six algorithms by achieving the maximum network coverage rate and the best node distribution uniformity. Moreover, the proposed algorithm was also demonstrated to have the fastest convergence speed and the highest stability in coverage optimization. As the number of sensor nodes increased, the OLMBES algorithm not only improved network coverage but also enhanced the uniformity of node distribution. However, for the other six algorithms, although the optimized network coverage rate increased with more nodes, the uniformity of node distribution was not improved. Finally, for different sizes of coverage areas, the simulation results also confirmed that the proposed OLMBES algorithm is highly adaptable and able to provide a high-quality network coverage control solution.

## 8. Conclusions

Coverage control is a fundamental and critical issue in WSN applications. In order to further improve the coverage performance of nodes to WSN, this paper proposes a multi-strategy bald eagle search algorithm with orthogonal learning embedded. The algorithm introduces Lévy flight, QRBL, and QI into the BES algorithm, which accelerate the convergence speed and improve the global search ability of the algorithm. When the fitness of the global optimal solution falls into stagnation during the iterative process of the algorithm, the OL update strategy is triggered to help the algorithm find a better position guidance vector, jumping out of the local optimal solution and enhancing the robustness of the algorithm.

The performance of the OLMBES algorithm is verified on CEC2014 benchmark functions. The proposed method is successfully applied to the wireless sensor network coverage optimization problem, and three sets of simulation experiments are set up to compare with the BES, GWO, WOA, SSA, BA, and CS algorithms. Although the proposed method has the same drawback in that the computational complexity is positively correlated with the grid point density as the centralized coverage optimization algorithm, it further improves the network coverage and node uniformity and has a faster convergence speed and greater robustness.

In future work, the energy consumption of sensor nodes will be comprehensively considered to extend the lifetime of the WSN while ensuring adequate coverage. For the purpose of reducing the computational complexity, future investigation will also focus on combining the centralized method and the distributed method to tackle the coverage optimization problem. Moreover, we wish to apply the proposed method in three-dimensional (3D) space and other actual scenes.

## Figures and Tables

**Table 1:** The pseudo-code of proposed OLMBES algorithm.

The pseudo-code of proposed OLMBES algorithm
Initialize the OLMBES parameters;
Randomly generate initial population;
**For** *i* = 1:*nPop*
Calculate the fitness of initial population;
**End For**
Q* = the optimal solution;
*Q^s^* = the second optimal solution;
*Q^t^* = the third optimal solution;
**While** (*iteration* ≤ *Maxgen)*
**Select space**
**For** (each individual *i* in the population)
Update the position using Equations (5) and (14);
$\mathrm{Evaluate}\;f(Q_{i}^{new})$, $f(Q_{i}^{levy})$, *f*(*Q_i_*) and choose the best individual as *Q_i_*;
**End For**
**Search in space**
**For** (each individual *i* in the population)
Update the position using Equations (6) and (19);
$\mathrm{Evaluate}\;f(Q_{i}^{new})$, $f(Q_{i}^{qr})$, *f*(*Q_i_*) and choose the best individual as *Q_i_*;
**End For**
**Swooping**
**For** (each individual *i* in the population)
*r* = randperm(*nPop*)
**If** *i* = *r*(1)
Update the position using Equation (10);
**else**
Update the position using Equation (20);
**End If**
$\mathrm{Evaluate}\;f(Q_{i}^{new})$, *f*(*Q_i_*) and choose the best individual as *Q_i_*;
**End For**
Update Q*, *Q^s^*, *Q^t^*;
**OL strategy**
**If** *stagnated_num* ≥ *limit*
$Q^{gv}=OED\left(Q^{*},Q^{s},Q_{i},\bar{Q_{i}}\right)$
Evaluate *f*(*Q^gv^*), *f*(Q*) and choose the best individual as Q^*^;
*stagnated_num* = 0;
**else**
*stagnated_num* = *stagnated_num +* 1;
**End If**
*iteration* = *iteration* + 1;
**End While**

**Table 2:** Comparative results of different algorithms on CEC2014 benchmark functions.

Problems	Statistics	OLMBES	OLMBES-1	OLMBES-2	OLMBES-3	BES	GWO	WOA	SSA	BA	CS
*F*1	Mean	**3.1820 × 10^3^**	9.7821 × 10^3^	8.2489 × 10^4^	1.7632 × 10^5^	4.5361 × 10^5^	7.5337 × 10^7^	1.3411 × 10^8^	1.9224 × 10^7^	1.3290 × 10^7^	4.3731 × 10^6^
	STD	**1.5479 × 10^3^**	2.0798 × 10^3^	4.6990 × 10^4^	1.6879 × 10^5^	2.6220 × 10^5^	4.1621 × 10^7^	4.8072 × 10^7^	6.0163 × 10^6^	1.3210 × 10^7^	4.3712 × 10^6^
*F*2	Mean	**1.8853 × 10^3^**	2.4708 × 10^3^	3.0579 × 10^3^	2.4789 × 10^3^	4.8839 × 10^3^	6.2216 × 10^9^	1.5911 × 10^9^	7.3036 × 10^3^	1.4007 × 10^6^	4.9723 × 10^7^
	STD	2.1328 × 10^3^	2.8302 × 10^3^	3.0495 × 10^3^	**2.1237 × 10^3^**	4.5945 × 10^3^	3.0028 × 10^9^	5.9508 × 10^8^	6.8858 × 10^3^	8.4116 × 10^5^	1.2827 × 10^7^
*F*3	Mean	**3.0650 × 10^2^**	3.0844 × 10^2^	3.0967 × 10^2^	1.3404 × 10^3^	1.5424 × 10^3^	6.8244 × 10^4^	1.0378 × 10^5^	5.7919 × 10^4^	3.0500 × 10^5^	7.9680 × 10^4^
	STD	4.0519 × 10^0^	**3.8738 × 10^0^**	4.5274 × 10^0^	7.3993 × 10^2^	1.0808 × 10^3^	1.3343 × 10^4^	2.3284 × 10^4^	1.2651 × 10^4^	1.9889 × 10^5^	1.0209 × 10^4^
*F*4	Mean	**4.1507 × 10^2^**	4.2460 × 10^2^	4.2762 × 10^2^	4.3389 × 10^2^	4.5305 × 10^2^	1.0290 × 10^3^	9.8126 × 10^2^	5.2079 × 10^2^	6.0023 × 10^2^	5.5509 × 10^2^
	STD	3.1426 × 10^1^	3.1414 × 10^1^	3.4590 × 10^1^	3.4787 × 10^1^	3.8879 × 10^1^	1.9751 × 10^2^	1.4742 × 10^2^	4.0292 × 10^2^	6.4912 × 10^1^	**1.8187 × 10^1^**
*F*5	Mean	5.2106 × 10^2^	5.2111 × 10^2^	5.2110 × 10^2^	5.2113 × 10^2^	5.2113 × 10^2^	5.2118 × 10^2^	5.2083 × 10^2^	**5.2004 × 10^2^**	5.2063 × 10^2^	5.2113 × 10^2^
	STD	4.8100 × 10^-2^	8.7600 × 10^-2^	1.3510 × 10^-1^	3.7600 × 10^-2^	5.0000 × 10^-2^	2.8400 × 10^-2^	**1.2600 × 10^-2^**	6.2100 × 10^-2^	6.4500 × 10^-2^	3.9500 × 10^-2^
*F*6	Mean	6.3176 × 10^2^	6.3537 × 10^2^	6.3421 × 10^2^	6.5592 × 10^2^	6.4438 × 10^2^	**6.3101 × 10^2^**	6.6874 × 10^2^	6.3961 × 10^2^	6.7121 × 10^2^	6.5426 × 10^2^
	STD	4.1231 × 10^0^	5.7097 × 10^0^	4.8988 × 10^0^	6.6622 × 10^0^	4.3224 × 10^0^	3.8579 × 10^0^	3.1133 × 10^0^	7.0864 × 10^0^	3.0445 × 10^0^	**1.3467 × 10^0^**
*F*7	Mean	**7.0000 × 10^2^**	7.0001 × 10^2^	7.0003 × 10^2^	7.0003 × 10^2^	7.0005 × 10^2^	7.5898 × 10^2^	7.1417 × 10^2^	**7.0000 × 10^2^**	7.0088 × 10^2^	7.0126 × 10^2^
	STD	1.1900 × 10^-2^	6.1000 × 10^-3^	**4.6000 × 10^-3^**	2.0750 × 10^-2^	2.3816 × 10^-2^	2.7691 × 10^1^	4.4903 × 10^1^	7.8000 × 10^-3^	8.0100 × 10^-2^	6.4000 × 10^-2^
*F*8	Mean	**8.8320 × 10^2^**	9.7830 × 10^2^	8.8875 × 10^2^	8.8872 × 10^2^	9.9456 × 10^2^	9.9537 × 10^2^	1.1582 × 10^3^	1.0818 × 10^3^	1.1124 × 10^3^	1.0606 × 10^3^
	STD	1.3435 × 10^1^	4.1773 × 10^1^	1.3954 × 10^1^	**1.2986 × 10^1^**	2.1363 × 10^1^	2.8809 × 10^1^	6.0159 × 10^1^	4.8441 × 10^1^	4.2866 × 10^1^	1.9962 × 10^1^
*F*9	Mean	**9.2532 × 10^2^**	1.1168 × 10^3^	1.1723 × 10^3^	1.2193 × 10^3^	1.2265 × 10^3^	1.0946 × 10^3^	1.4012 × 10^3^	1.1713 × 10^3^	1.2925 × 10^3^	1.2700 × 10^3^
	STD	8.9037 × 10^1^	4.8512 × 10^1^	1.0096 × 10^2^	7.0924 × 10^1^	3.9698 × 10^1^	2.8493 × 10^1^	9.0448 × 10^1^	6.2147 × 10^1^	7.4593 × 10^1^	**2.1871 × 10^1^**
*F*10	Mean	**2.5973 × 10^3^**	2.7618 × 10^3^	2.9140 × 10^3^	2.9511 × 10^3^	6.2658 × 10^3^	6.7547 × 10^3^	9.5217 × 10^3^	8.1038 × 10^3^	8.3857 × 10^3^	7.0936 × 10^3^
	STD	5.4336 × 10^2^	7.3431 × 10^2^	1.8091 × 10^3^	7.5514 × 10^2^	6.6441 × 10^2^	8.1849 × 10^2^	1.4181 × 10^3^	9.4484 × 10^2^	8.4668 × 10^2^	**2.5924 × 10^2^**
*F*11	Mean	**2.0335 × 10^3^**	5.2370 × 10^3^	6.2135 × 10^3^	8.0269 × 10^3^	8.3906 × 10^3^	7.2466 × 10^3^	1.0971 × 10^4^	7.8590 × 10^3^	8.9508 × 10^3^	9.0488 × 10^3^
	STD	**9.3474 × 10^1^**	5.6362 × 10^2^	6.9241 × 10^2^	9.3474 × 10^2^	2.5459 × 10^3^	9.2996 × 10^2^	1.0103 × 10^3^	1.1171 × 10^3^	9.6015 × 10^2^	3.3310 × 10^2^
*F*12	Mean	**1.2002 × 10^3^**	1.2003 × 10^3^	1.2004 × 10^3^	1.2022 × 10^3^	1.2024 × 10^3^	7.5337 × 10^7^	1.3411 × 10^8^	1.9224 × 10^7^	2.0790 × 10^7^	3.1631 × 10^7^
	STD	**9.8200 × 10^-2^**	1.4300 × 10^-1^	1.3970 × 10^-1^	9.1830 × 10^-1^	1.0530 × 10^0^	4.1621 × 10^7^	4.8072 × 10^7^	6.0163 × 10^6^	1.3210 × 10^7^	4.3712 × 10^6^
*F*13	Mean	**1.3001 × 10^3^**	1.3004 × 10^3^	1.3005 × 10^3^	1.3005 × 10^3^	1.3007 × 10^3^	1.3007 × 10^3^	1.3006 × 10^3^	1.3006 × 10^3^	1.3005 × 10^3^	1.3004 × 10^3^
	STD	8.3900 × 10^-2^	1.0010 × 10^-1^	9.9200 × 10^-2^	8.7300 × 10^-2^	1.2570 × 10^-1^	8.4800 × 10^-2^	9.3300 × 10^-2^	1.2410 × 10^-1^	8.9800 × 10^-2^	**4.2300 × 10^-2^**
*F*14	Mean	**1.4000 × 10^3^**	**1.4000 × 10^3^**	1.4003 × 10^3^	1.4003 × 10^3^	1.4004 × 10^3^	1.4109 × 10^3^	1.4004 × 10^3^	1.4006 × 10^3^	1.4003 × 10^3^	1.4003 × 10^3^
	STD	4.8800 × 10^-2^	1.5769 × 10^-1^	1.1590 × 10^-1^	1.2120 × 10^-1^	1.1279 × 10^-1^	9.0474 × 10^0^	1.6710 × 10^-1^	2.6040 × 10^-1^	7.6800 × 10^-2^	**2.0600 × 10^-2^**
*F*15	Mean	**1.5109 × 10^3^**	1.5245 × 10^3^	1.5277 × 10^3^	1.5274 × 10^3^	1.5374 × 10^3^	2.6384 × 10^3^	2.9205 × 10^3^	1.5220 × 10^3^	1.9248 × 10^3^	1.5464 × 10^3^
	STD	**2.4730 × 10^0^**	1.3852 × 10^1^	7.7099 × 10^0^	5.9136 × 10^0^	1.3941 × 10^1^	2.1549 × 10^3^	1.4458 × 10^3^	6.4046 × 10^0^	8.5514 × 10^1^	3.7090 × 10^0^
*F*16	Mean	**1.6115 × 10^3^**	1.6206 × 10^3^	1.6219 × 10^3^	1.6219 × 10^3^	1.6224 × 10^3^	1.6210 × 10^3^	1.6225 × 10^3^	1.6211 × 10^3^	1.6227 × 10^3^	1.6223 × 10^3^
	STD	5.7080 × 10^-1^	5.4910 × 10^-1^	7.6120 × 10^-1^	4.8754 × 10^-1^	6.2289 × 10^-1^	1.0547 × 10^0^	4.6549 × 10^-1^	6.7810 × 10^-1^	6.1230 × 10^-1^	**1.9115 × 10^-1^**
*F*17	Mean	**2.2612 × 10^4^**	2.3816 × 10^4^	2.6387 × 10^4^	3.2701 × 10^4^	3.1594 × 10^4^	4.2176 × 10^6^	6.1585 × 10^7^	1.8051 × 10^6^	2.0263 × 10^6^	2.9514 × 10^6^
	STD	**9.1357 × 10^3^**	2.3361 × 10^4^	1.2612 × 10^4^	1.3041 × 10^4^	2.2971 × 10^4^	3.1345 × 10^6^	3.4385 × 10^7^	1.0147 × 10^6^	1.9203 × 10^6^	3.4245 × 10^5^
*F*18	Mean	**2.5416 × 10^3^**	3.6805 × 10^3^	3.7324 × 10^3^	3.7618 × 10^3^	4.1837 × 10^3^	1.7814 × 10^7^	7.7050 × 10^5^	4.1228 × 10^3^	3.4029 × 10^4^	2.2305 × 10^4^
	STD	**6.2703 × 10^2^**	1.6624 × 10^3^	1.7083 × 10^3^	1.8009 × 10^3^	1.6298 × 10^3^	4.0682 × 10^7^	1.4604 × 10^6^	1.5807 × 10^7^	8.1861 × 10^3^	2.6244 × 10^3^
*F*19	Mean	**1.9125 × 10^3^**	1.9166 × 10^3^	1.9325 × 10^3^	1.9211 × 10^3^	1.9241 × 10^3^	1.9831 × 10^3^	2.0470 × 10^3^	1.9380 × 10^3^	1.9729 × 10^3^	1.9335 × 10^3^
	STD	3.3048 × 10^0^	1.2003 × 10^1^	3.1423 × 10^0^	**2.3881 × 10^0^**	1.1976 × 10^1^	2.7042 × 10^1^	7.3105 × 10^1^	1.5776 × 10^1^	3.2126 × 10^1^	3.9217 × 10^0^
*F*20	Mean	**2.3175 × 10^3^**	2.3671 × 10^3^	2.4072 × 10^3^	2.4251 × 10^3^	2.6052 × 10^3^	2.5660 × 10^4^	2.5168 × 10^5^	2.5270 × 10^4^	1.0789 × 10^5^	2.6022 × 10^4^
	STD	**7.4845 × 10^1^**	1.1310 × 10^2^	1.3901 × 10^2^	1.1199 × 10^2^	2.4131 × 10^2^	8.4631 × 10^3^	2.1040 × 10^5^	1.1150 × 10^4^	6.9083 × 10^4^	6.9989 × 10^3^
*F*21	Mean	**1.1428 × 10^4^**	1.2980 × 10^4^	2.2481 × 10^4^	1.2980 × 10^4^	1.7789 × 10^4^	3.2236 × 10^6^	1.2556 × 10^7^	8.1890 × 10^5^	1.1928 × 10^6^	5.3020 × 10^5^
	STD	**9.7132 × 10^3^**	1.7688 × 10^4^	1.2557 × 10^4^	3.1000 × 10^4^	1.1035 × 10^4^	2.4071 × 10^6^	6.0423 × 10^6^	4.6961 × 10^5^	2.0040 × 10^6^	1.5194 × 10^5^
*F*22	Mean	2.7534 × 10^3^	**2.5182 × 10^3^**	3.0584 × 10^3^	3.2347 × 10^3^	3.0276 × 10^3^	3.0275 × 10^3^	4.3698 × 10^3^	3.4246 × 10^3^	4.4362 × 10^3^	3.2100 × 10^3^
	STD	**1.1710 × 10^2^**	1.3869 × 10^2^	2.8654 × 10^2^	1.8543 × 10^2^	2.6189 × 10^2^	2.3956 × 10^2^	5.5036 × 10^2^	2.9716 × 10^2^	4.2964 × 10^2^	1.4045 × 10^2^
*F*23	Mean	**2.5072 × 10^3^**	**2.5072 × 10^3^**	2.5144 × 10^3^	2.5144 × 10^3^	2.5216 × 10^3^	2.7265 × 10^3^	2.7726 × 10^3^	2.6656 × 10^3^	2.6600 × 10^3^	2.6444 × 10^3^
	STD	3.2134 × 10^1^	3.2161 × 10^1^	4.4262 × 10^1^	4.4304 × 10^1^	5.2756 × 10^1^	2.2742 × 10^1^	2.5022 × 10^1^	6.1307 × 10^0^	9.8568 × 10^0^	**8.6800 × 10^-2^**
*F*24	Mean	**2.6000 × 10^3^**	**2.6000 × 10^3^**	2.6013 × 10^3^	2.6047 × 10^3^	2.6039 × 10^3^	**2.6000 × 10^3^**	**2.6006 × 10^3^**	2.6888 × 10^3^	2.7448 × 10^3^	2.6900 × 10^3^
	STD	**3.7518 × 10^-7^**	1.8241 × 10^-6^	20.294 × 10^-6^	1.4201 × 10^-6^	1.6743 × 10^-6^	6.3000 × 10^-3^	1.0636 × 10^0^	1.0261 × 10^1^	2.6492 × 10^1^	1.6503 × 10^0^
*F*25	Mean	**2.7000 × 10^3^**	**2.7000 × 10^3^**	**2.7000 × 10^3^**	**2.7000 × 10^3^**	**2.7000 × 10^3^**	2.7278 × 10^3^	2.7061 × 10^3^	2.7261 × 10^3^	2.7479 × 10^3^	2.7272 × 10^3^
	STD	**0**	**0**	**0**	**0**	**0**	7.0474 × 10^0^	1.8895 × 10^1^	6.0762 × 10^0^	1.2458 × 10^1^	2.3426 × 10^0^
*F*26	Mean	**2.7001 × 10^3^**	**2.7001 × 10^3^**	2.7005 × 10^3^	2.7800 × 10^3^	2.7750 × 10^3^	2.7923 × 10^3^	2.7004 × 10^3^	2.7006 × 10^3^	2.7275 × 10^3^	2.7004 × 10^3^
	STD	1.5431 × 10^2^	1.7264 × 10^2^	1.8705 × 10^2^	1.8879 × 10^2^	2.0742 × 10^2^	4.6954 × 10^1^	9.4800 × 10^-2^	1.1860 × 10^-1^	7.3204 × 10^1^	**3.4300 × 10^-2^**
*F*27	Mean	**2.9000 × 10^3^**	3.1341 × 10^3^	3.8265 × 10^3^	3.9540 × 10^3^	3.7809 × 10^3^	3.7615 × 10^3^	4.8413 × 10^3^	4.0325 × 10^3^	4.9454 × 10^3^	3.5695 × 10^3^
	STD	9.8316 × 10^1^	1.0943 × 10^2^	3.0914 × 10^2^	4.0366 × 10^2^	1.7887 × 10^2^	9.7524 × 10^1^	1.2738 × 10^2^	1.6164 × 10^2^	**7.7465 × 10^1^**	3.2127 × 10^2^
*F*28	Mean	4.4248 × 10^3^	**4.2575 × 10^3^**	4.4560 × 10^3^	4.6795 × 10^3^	4.6716 × 10^3^	5.0013 × 10^3^	8.3661 × 10^3^	4.9647 × 10^3^	8.5051 × 10^3^	4.2812 × 10^3^
	STD	3.1162 × 10^2^	1.0867 × 10^3^	7.1191 × 10^2^	4.0583 × 10^2^	4.3825 × 10^2^	6.3735 × 10^2^	1.6951 × 10^3^	6.8043 × 10^2^	2.1457 × 10^3^	**6.6154 × 10^1^**
*F*29	Mean	1.7611 × 10^5^	1.8690 × 10^5^	2.0766 × 10^5^	2.5024 × 10^5^	2.8723 × 10^5^	6.3729 × 10^6^	4.4366 × 10^7^	5.0505 × 10^7^	**1.4954 × 10^5^**	2.1996 × 10^5^
	STD	1.4539 × 10^5^	2.5081 × 10^5^	1.0759 × 10^5^	2.3749 × 10^5^	1.8872 × 10^5^	9.8300 × 10^6^	2.7418 × 10^7^	5.9499 × 10^7^	1.3191 × 10^5^	**6.4697 × 10^4^**
*F*30	Mean	1.3793 × 10^4^	1.3440 × 10^4^	**1.1795 × 10^4^**	1.5423 × 10^4^	1.4625 × 10^4^	1.7032 × 10^5^	3.0252 × 10^5^	7.2206 × 10^4^	3.2486 × 10^4^	3.1718 × 10^4^
	STD	1.4711 × 10^3^	1.6530 × 10^3^	**9.5112 × 10^2^**	1.6869 × 10^3^	1.5527 × 10^3^	6.6880 × 10^4^	2.3657 × 10^5^	3.8024 × 10^4^	2.6221 × 10^4^	4.5067 × 10^3^

**Table 3:** Parameter settings of algorithms.

Algorithm	Parameter Settings
OLMBES	*δ* = 1.5, *α* = 10, *R* = 1.5, *c_1_* = *c_2_* = 2, *k* = 10, *limit* = 5
BES	*δ* = 1.5, *α* = 10, *R* = 1.5, *c_1_* = *c_2_* = 2
GWO	*a_ini_* = 2
WOA	*a_ini_* = 2
SSA	*c*_1_, *c*_2_∈ [0, 1]
BA	*A_o_* = 0.5, *p_r_* = 0.5, *f_min_* = 0, *f_max_* = 2
CS	*p_a_* = 0.25

**Table 4:** Coverage performance indicators of algorithms.

Algorithm	Coverage Rate	Uniformity
OLMBES	**0.9602**	**0.3485**
BES	0.9377	0.5255
GWO	0.9333	0.5523
WOA	0.9186	0.5974
SSA	0.9348	0.5274
BA	0.9371	0.5263
CS	0.9263	0.5826

**Table 5:** Coverage rates with different numbers of nodes.

Number of Nodes	24	26	28	30	32
OLMBES	**0.8890**	**0.9190**	**0.9421**	**0.9602**	**0.9742**
BES	0.8747	0.9027	0.9245	0.9377	0.9562
GWO	0.8676	0.8806	0.9174	0.9333	0.9339
WOA	0.8477	0.8807	0.9006	0.9186	0.9387
SSA	0.8731	0.9018	0.9191	0.9348	0.9547
BA	0.8726	0.9021	0.9233	0.9371	0.9537
CS	0.8522	0.8812	0.9065	0.9263	0.9443

**Table 6:** Uniformity with different numbers of nodes.

Number of Nodes	24	26	28	30	32
OLMBES	**0.4045**	**0.4066**	**0.3809**	**0.3485**	**0.3201**
BES	0.4522	0.4943	0.4816	0.5255	0.4838
GWO	0.4633	0.5447	0.5392	0.5523	0.6153
WOA	0.6001	0.5943	0.6031	0.5974	0.5982
SSA	0.4680	0.4825	0.4984	0.5274	0.4865
BA	0.4739	0.4791	0.4939	0.5263	0.4912
CS	0.6193	0.5895	0.6367	0.5826	0.5972

**Table 7:** Parameter settings of different surveillance region sizes.

Surveillance Area Size	Sensing Radius	Communication Radius
20 m × 20 m	2.5 m	5 m
40 m × 40 m	5 m	10 m
60 m × 60 m	7.5 m	15 m

**Table 8:** Coverage rates of different surveillance region sizes.

Surveillance Area Size	20 m × 20 m	40 m × 40 m	60 m × 60 m
OLMBES	**0.9602**	**0.9644**	**0.9624**
BES	0.9377	0.9462	0.9331
GWO	0.9333	0.9410	0.9344
WOA	0.9186	0.9251	0.9206
SSA	0.9348	0.9375	0.9341
BA	0.9371	0.9408	0.9366
CS	0.9263	0.9330	0.9309

**Table 9:** Uniformity with different surveillance region sizes.

Surveillance Area Size	20 m × 20 m	40 m × 40 m	60 m × 60 m
OLMBES	**0.3485**	**0.6567**	**1.0002**
BES	0.5255	0.9461	1.7508
GWO	0.5523	0.9981	1.5662
WOA	0.5974	1.2009	1.8364
SSA	0.5274	1.0061	1.5945
BA	0.5263	0.9821	1.5638
CS	0.5826	1.1601	1.8108

## Data Availability

The data used to support the findings of this study are available from the corresponding author upon request.
